# Effectiveness and mechanisms of lymphocytes at different time points in predicting consolidation immunotherapy following adaptive chemoradiotherapy in locally advanced non-small cell lung cancer

**DOI:** 10.3389/fonc.2025.1683430

**Published:** 2026-01-09

**Authors:** Zhen wei Sun, Jiayi Duan, Zhaohao Zhang, Meng Chen, Dandan Zhou, Liqiao Hou, Pingjun Gu, Jian Zhu, Haihua Yang, Suna Zhou

**Affiliations:** 1 Taizhou Hospital of Zhejiang Province, Shaoxing University, Taizhou, Zhejiang, China; 2 Key Laboratory of Radiation Oncology of Taizhou, Radiation Oncology Institute of Enze Medical Health Academy, Department of Radiation Oncology, Taizhou Hospital Affiliated to Wenzhou Medical University, Taizhou, Zhejiang, China; 3 Taizhou Enze Hospital affiliated to Hangzhou Medical College, Taizhou, Zhejiang, China

**Keywords:** lymphocytes, lung cancer, radiotherapy, consolidation immunotherapy, predictive markers

## Abstract

**Objective:**

To investigate the prognostic value of lymphocytes and their subsets at different stages of chemoradiotherapy and consolidation immunotherapy in patients with locally advanced non-small cell lung cancer (NSCLC).

**Methods:**

This retrospective analysis enrolled 139 patients with stage III NSCLC (median age 69 years; 95% male; 84.17% squamous cell carcinoma) who received adaptive chemoradiotherapy (CRT) and consolidation anti-PD-1 therapy (sintilimab). Paired samples t-tests were performed to evaluate differences in absolute lymphocyte counts (ALCs) at three time points: before radiotherapy, at the 20th fraction of radiotherapy, and 1 month after radiotherapy. Additionally, paired t-tests were used to compare lymphocyte subsets between the pre-radiotherapy period and 1 month post-radiotherapy. Univariate and multivariate Cox proportional hazards regression analyses were conducted to identify factors influencing progression-free survival (PFS) and overall survival (OS). The Kaplan–Meier analysis was employed to assess PFS and OS in patients stratified according to independent predictive factors associated with OS.

**Results:**

Statistically significant differences were observed in ALCs among the three time points before radiotherapy, at the 20th fraction of radiotherapy, and 1 month after radiotherapy (p < 0.05). Univariate and multivariate analyses identified ALCs at 1 month post-radiotherapy, ALC decrease (defined as the difference between pre-radiotherapy and the 20th fraction of adaptive radiotherapy), and ALC increase (defined as the difference between the 20th fraction and pre-radiotherapy) as independent predictors of OS (p < 0.05). The Kaplan–Meier curves demonstrated that patients with ALCs > 1.015 × 10^9^/L at 1 month after radiotherapy, an ALC decrease > 0.71 × 10^9^/L, or an ALC increase > 0.305 × 10^9^/L had significantly longer OS. Significant differences were also observed in CD4+ and CD8+ counts, as well as the CD8/CD4 ratio, between pre-radiotherapy and 1 month post-radiotherapy (p < 0.05). The Kaplan–Meier analysis further showed that patients with higher CD8+ T-cell counts at 1 month post-radiotherapy had significantly longer OS (p < 0.05).

**Conclusion:**

In patients with locally advanced NSCLC receiving chemoradiotherapy followed by consolidation immunotherapy, higher ALCs and elevated CD8+ T-cell counts at 1 month post-chemoradiotherapy are associated with improved overall survival.

## Introduction

Lung cancer is predominantly driven by non-small cell lung cancer (NSCLC), which accounts for 80%–85% of all cases (1). Approximately 20%–30% of patients are diagnosed at stage III, of whom 60%–90% present with unresectable disease (2–4). For patients with unresectable locally advanced NSCLC (LA-NSCLC), concurrent chemoradiotherapy (cCRT) has long been the standard treatment. However, the long-term efficacy of cCRT remains unsatisfactory, with 5-year survival rates typically ranging from 15% to 30% (2–5). Therapeutic options for LA-NSCLC have expanded significantly since the publication of the PACIFIC trial in 2017, which reported practice-changing results (6). This randomized, double-blind, placebo-controlled Phase III study enrolled patients with unresectable stage III NSCLC and evaluated the use of durvalumab, a programmed death-ligand 1 (PD-L1) immune checkpoint inhibitor (ICI), as consolidation therapy following curative-intent cCRT (7). With a median follow-up of 34.2 months, the most recent survival analyses demonstrated that consolidation durvalumab achieved a 5-year overall survival (OS) rate of 42.9% and a progression-free survival (PFS) rate of 33.1% (8). The PACIFIC trial established that consolidation durvalumab following CRT (the PACIFIC regimen) provides substantial and durable survival benefits compared to CRT alone, further solidifying its role as the new standard of care in this setting (6, 8–12). Moreover, the global multicenter Phase II KEYNOTE-799 study, an open-label, multi-cohort, non-randomized trial, demonstrated that pembrolizumab combined with concurrent chemoradiotherapy (CRT) exhibited promising anti-tumor activity and manageable safety in patients with newly diagnosed, locally advanced stage III NSCLC.

Although the PACIFIC regimen has improved outcomes in NSCLC, a subset of patients still exhibits poor responses. The use of biomarkers to predict immunotherapy efficacy has gained increasing attention. Key markers include PD-L1 expression, blood-based tumor mutational burden (bTMB), microsatellite instability (MSI), DNA damage repair (DDR) mechanisms, and tumor-infiltrating lymphocytes (TILs) (13, 14). However, due to changes in the immune microenvironment and biomarker profiles following radiotherapy, it is necessary to further investigate predictive markers for immune consolidation at different stages. Invasive post-radiotherapy biopsies limit the feasibility of assessing certain markers, whereas blood lymphocyte measurements appear to be a more practical and minimally invasive alternative. Beyond tissue-based biomarkers, peripheral blood parameters offer a dynamic and accessible window into the host’s immune status. The absolute lymphocyte count (ALC) has emerged as a key systemic immune biomarker, where baseline levels and on-treatment kinetics are prognostic across stages of NSCLC treated with immunotherapy. For instance, in patients with advanced NSCLC receiving first-line pembrolizumab plus chemotherapy, a higher baseline ALC was independently associated with significantly improved overall survival (15). Furthermore, in the context of locally advanced disease, the severity of radiation-induced lymphopenia during chemo-radiation has been consistently linked to poorer survival outcomes, underscoring the critical role of preserving lymphocyte reserves for optimal therapeutic efficacy (16). Thus, identifying more effective approaches to predict immunotherapy efficacy is of paramount importance.

This study retrospectively analyzed the predictive value of peripheral blood lymphocytes at multiple time points for outcomes of consolidation immunotherapy following adaptive CRT in NSCLC. Furthermore, by examining changes in lymphocyte subsets before and after chemoradiotherapy, the study preliminarily elucidated the mechanisms by which alterations in the systemic immune microenvironment post-radiotherapy may predict treatment efficacy.

## Patients and methods

### Patient selection

This study retrospectively reviewed the medical records of NSCLC patients treated at Taizhou Hospital, Zhejiang Province, China, from December 1, 2018, to July 31, 2024. The inclusion criteria were as follows: 1) age 18–80 years; 2) complete electronic health records, including ALCs; 3) pathologically confirmed NSCLC; 4) clinically unresectable stage III disease; 5) Eastern Cooperative Oncology Group (ECOG) performance status of 0 or 1 at baseline, with a projected survival of at least 6 months; and (6) receipt of at least two cycles of anti-PD-1 inhibitor therapy.

Patients were excluded if they met any of the following criteria: 1) uncertain NSCLC diagnosis; 2) presence of other malignancies; 3) incomplete clinical data; 4) underlying conditions such as acute infections, hematologic disorders, autoimmune diseases, pregnancy, or lactation; and 5) genetic alterations in the epidermal growth factor receptor (EGFR) or anaplastic lymphoma kinase (ALK) that could affect treatment outcomes. The primary study endpoints were OS and PFS. The therapeutic efficacy of anti-PD-1 inhibitors was assessed using CT or magnetic resonance imaging (MRI), according to the Response Evaluation Criteria in Solid Tumors (RECIST v1.1) (17).

### Data collection

Patient medical records were reviewed to collect demographic and clinical data, including age, gender, smoking history, histological type, lymph node metastasis, clinical stage, and treatment modality (concurrent or sequential chemoradiotherapy). Data on PFS, OS, and ALCs were collected at three time points: before radiotherapy, during radiotherapy (20th fraction), and 1 month after radiotherapy (prior to immunotherapy). Immunological parameters, including CD4+ T-cell counts, CD8+ T-cell counts, and the CD8/CD4 ratio, were also collected before radiotherapy and 1 month post-radiotherapy. Tumor staging was determined according to the eighth edition of the American Joint Committee on Cancer (AJCC) staging system.

### Treatment

All patients received radical simultaneous integrated boost intensity-modulated radiotherapy (SIB-IMRT). Target delineation was performed using contrast-enhanced CT or 4D-CT (slice thickness ≤5 mm): the gross tumor volume (GTV; primary tumor plus nodes ≥1 cm) was expanded by 0.5–1 cm to form the planning GTV (PGTV); the clinical target volume (CTV) included involved nodes only, without elective nodal irradiation (ENI); the planning target volume (PTV) added 0.5–1.5-cm margins to the CTV to account for motion and setup errors, quantified using 4D-CT. Initial and mid-treatment planning were based on these datasets. PET/CT was occasionally used but not routinely for delineation. Megavoltage cone-beam CT (MV/CBCT) imaging was employed to minimize geometric errors. Dose prescription: the PGTV received 2.14–2.2 Gy × 20 fractions (total 42.8–44 Gy), while the PTV received 54 Gy in 30 fractions (1.8 Gy/fraction), followed by a sequential PGTV boost to a total dose of 64–66 Gy. Plan adaptation was performed after 20 fractions via repeat CT simulation. Concurrent platinum-based doublet chemotherapy was preferred, with sequential chemotherapy or radiotherapy alone administered in cases of intolerance. Organs-at-risk (OAR) constraints were as follows: lung V20 < 35%, esophageal mean dose < 34 Gy, and heart V30 < 40% (18). All patients subsequently received consolidation immunotherapy with intravenous sintilimab at a dose of 200 mg every 3 weeks (19).

### Statistical analysis

OS was the primary endpoint of this study, and PFS was the secondary endpoint. Descriptive statistics were used to summarize patient characteristics. PFS was defined as the time from the first dose of immunotherapy to tumor recurrence, death from any cause, or censoring at the last follow-up. OS was defined as the time from the initiation of immunotherapy to death or the end of the follow-up period. To quantify lymphocyte dynamics, two key variables were defined: 1) “ALC decrease” as the reduction from pre-radiotherapy to the 20th fraction, quantifying treatment-induced lymphodepletion, and 2) “ALC increase” as the rise from the 20th fraction to 1 month post-radiotherapy, representing early recovery. Paired samples t-tests were used to assess changes in absolute lymphocyte counts at different time points. Univariate and multivariate Cox proportional hazards regression analyses were performed to examine the associations between lymphocyte levels at different periods and PFS and OS. Variables with p < 0.05 in univariate analysis were included in multivariate models to control for confounding factors and assess independent effects. The receiver operating characteristic (ROC) curve was used to determine optimal cutoff values for lymphocytes at different time points. Continuous variables are presented as mean ± standard deviation or median values, and categorical variables are expressed as frequencies and percentages. The chi-square test was employed to compare categorical variables between groups. The Kaplan–Meier survival analysis was used to evaluate PFS and OS. Statistical significance was set at p < 0.05. Data analyses were conducted using SPSS version 25.0 (Armonk, NY, USA) and GraphPad Prism version 8.0 (San Diego, CA, USA).

## Results

### Patient characteristics

A total of 139 NSCLC patients were enrolled according to the inclusion criteria. The median age was 69 years (range, 48–80 years). There were 132 men (95.0%), reflecting regional smoking patterns in Eastern China and potential institutional referral biases. A history of smoking was reported in 108 patients (70.5%). Histologically, 117 cases (84.17%) were squamous carcinoma, 13 cases (9.35%) were adenocarcinoma, and nine cases (6.48%) were other types, including large cell carcinoma and adenosquamous carcinoma. The ECOG performance status was 0 in 102 patients (73.38%). According to TNM staging, 48 patients (34.53%) were stage IIIA, 69 patients (49.64%) were stage IIIB, and 22 patients (15.83%) were stage IIIC. Regarding treatment, 76 patients (54.68%) received sequential chemoradiotherapy, and 63 patients (45.32%) received concurrent CRT. Baseline characteristics are summarized in **Table 1**.

**Table 1:** Baseline characteristics of patients.

Variables		Total (n = 139)
Age (year)		69.00 (48, 80)
Gender n(%)	Female	7 (5.04)
	Male	132 (94.96)
ECOG n(%)	0	102 (73.38)
	1	37 (26.62)
Smoking n(%)	YES	98 (70.5)
	NO	41 (29.5)
T n(%)	1	16 (11.51)
	2	31 (22.30)
	3	24 (17.27)
	4	68 (48.92)
N n(%)	1	20 (14.39)
	2	78 (56.12)
	3	41 (29.50)
Stage n(%)	IIIA	48 (34.53)
	IIIB	69 (49.64)
	IIIC	22 (15.83)
Pathologic n(%)	Squamous	117 (84.17)
	Adenocarcinoma	13 (9.35)
	Other	9 (6.47)
Therapy Method n(%)	Concurrent Chemoradiotherapy	63 (45.32)
	Sequential Chemoradiotherapy	76 (54.68)
ALCS before RT (Mean±SD)		1.39 ± 0.52
ALCS of 20^th^ fraction during RT (Mean±SD)		0.61 ± 0.80
ALCS of 1month after RT (Mean±SD)		1.10 ± 0.52
Decreased lymphocytes (Mean±SD)		0.78 ± 0.94
Elevated lymphocytes (Mean±SD)		0.49 ± 0.92

ECOG, The Eastern Cooperative Oncology Group Performance Status (ECOG) score; ALCS, absolute lymphocyte counts; RT, radiotherapy; SD, standard deviation.

### Changes in ALCs at different time points

**Figure 1:**
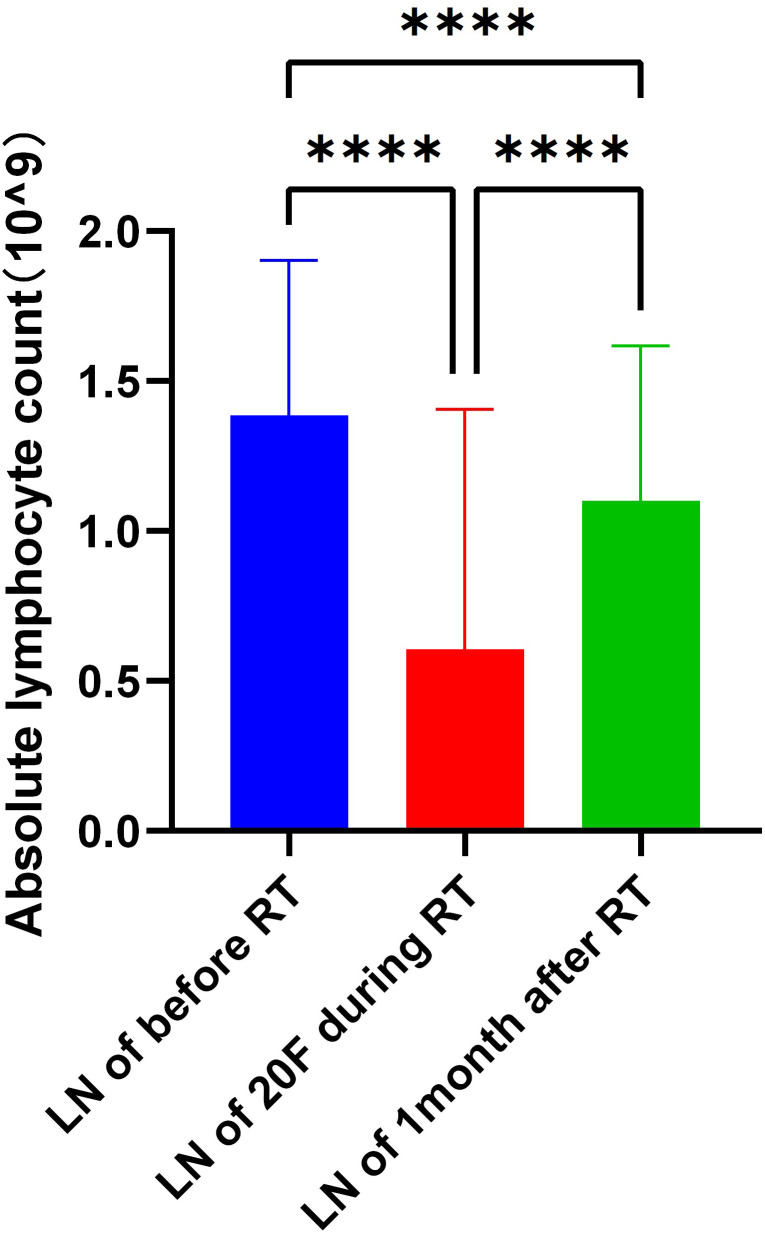
Changes in absolute lymphocyte counts at different time points. Absolute lymphocyte counts (ALCs) were measured before radiotherapy, at the 20th fraction of radiotherapy, and at 1 month following radiotherapy. Data are presented as mean ± standard deviation. ****p < 0.0001, paired samples t-test.

The ALCs were 1.39 ± 0.52 × 10^9^/L before radiotherapy, 0.61 ± 0.80 × 10^9^/L at the 20th fraction of radiotherapy, and 1.10 ± 0.52 × 10^9^/L 1 month after radiotherapy. The decrease in ALCs from before radiotherapy to the 20th fraction was 0.78 ± 0.94 × 10^9^/L, and the subsequent increase from the 20th fraction to 1 month post-radiotherapy was 0.49 ± 0.92 × 10^9^/L. Significant differences were observed for both the ALC decrease from baseline to the 20th fraction and the increase from the 20th fraction to 1 month post-radiotherapy (p < 0.05) (**Figure 1**).

### Correlation of ALCs with PFS

**Figure 2:**
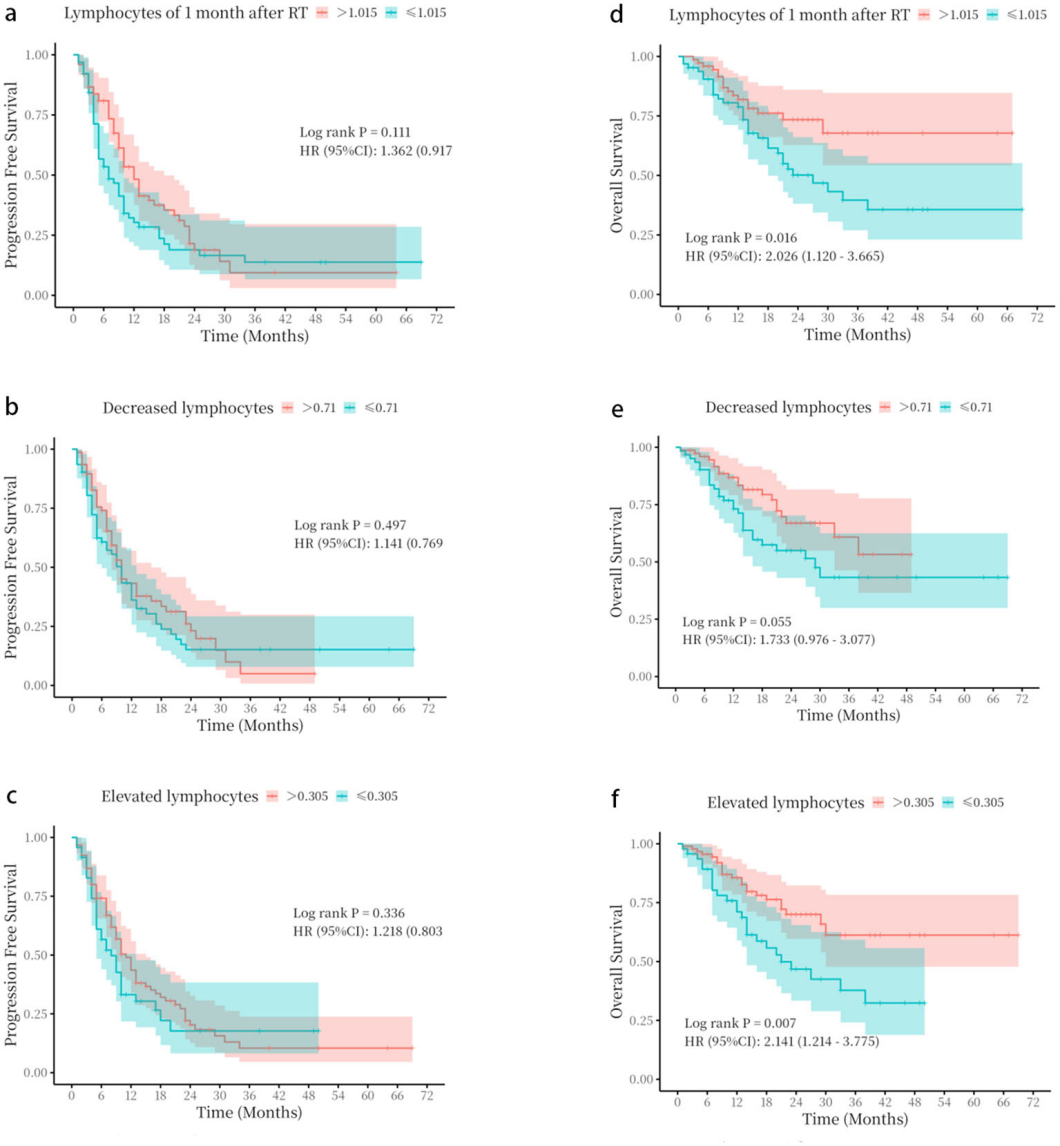
Kaplan–Meier survival curves based on absolute lymphocyte count parameters. **(a–c)** progression-free survival (PFS) stratified by **(a)** absolute lymphocyte count (ALC) at 1 month following radiotherapy, **(b)** ALC decrease, and **(c)** ALC increase. **(d–f)** Overall survival (OS) stratified by **(d)** ALC at 1 month following radiotherapy, **(e)** ALC decrease, and **(f)** ALC increase. The optimal cut-off values for stratification were determined by receiver operating characteristic (ROC) curve analysis. p-Values were calculated using the log-rank test.

Univariate analysis of variables, including treatment method, gender, age, smoking status, TNM stage, ECOG score, histological type, ALC before radiotherapy, ALC at the 20th fraction, ALC at 1 month post-radiotherapy, ALC decrease, and ALC increase, revealed no significant associations with PFS (**Supplementary Table 1**). In addition, patients with ALCs > 1.015 × 10^9^/L at 1 month after radiotherapy, ALC decrease > 0.71 × 10^9^/L, or ALC increase > 0.305 × 10^9^/L showed no significant difference in PFS (p > 0.05) (**Figure 2**).

### Correlation between ALCs and OS

As shown in **Table 2**, univariate analysis revealed that the ALCs at 1 month following radiotherapy, and the decrease and increase in ALCs were correlated with enhanced OS (p < 0.045; p = 0.038; p = 0.006). Multivariate analysis showed that the ALCs at 1 month after radiotherapy, decreased lymphocytes, and elevated lymphocytes could serve as independent predictors for significantly improved OS (OR = 0.44, 95% CI: 0.20–0.97, p = 0.041; OR = 0.46, 95% CI: 0.22–0.97, p = 0.041; OR = 0.33, 95% CI: 0.15–0.72, p = 0.005).

**Table 2:** Univariate and multivariate cox analysis was used to analyze overall survival.

Variables		Univariate analysis		Multivariate analysis	
		*P*	OR (95%CI)	*P*	OR (95%CI)
Age		0.080	0.97 (0.93 ~ 1.00)		
Gender	Male		1.00 (Reference)		
	Female	0.636	1.45 (0.31 ~ 6.76)		
ECOG	0		1.00 (Reference)		
	1	0.265	0.62 (0.27 ~ 1.43)		
Smoking	No		1.00 (Reference)		
	Yes	0.219	1.65 (0.74 ~ 3.69)		
T	1		1.00 (Reference)		
	2	0.892	0.92 (0.26 ~ 3.20)		
	3	0.787	0.83 (0.22 ~ 3.12)		
	4	0.781	0.85 (0.28 ~ 2.64)		
N	1		1.00 (Reference)		
	2	0.434	0.67 (0.24 ~ 1.84)		
	3	0.942	0.96 (0.32 ~ 2.86)		
Stage	IIIA		1.00 (Reference)		1.00 (Reference)
	IIIB	0.747	1.14 (0.52 ~ 2.47)	0.574	1.26 (0.56 ~ 2.84)
	IIIC	0.900	0.93 (0.32 ~ 2.75)	0.930	0.95 (0.30 ~ 2.98)
Pathologic	Squamous		1.00 (Reference)		
	Adenocarcinoma	0.549	0.58 (0.10 ~ 3.40)		
	Other	0.361	0.58 (0.18 ~ 1.85)		
Therapy Method	Concurrent Chemoradiotherapy		1.00 (Reference)		1.00 (Reference)
	Sequential Chemoradiotherapy	0.787	1.10 (0.55 ~ 2.23)	0.480	1.31 (0.62 ~ 2.76)
ALCS before RT		0.255	0.66 (0.33 ~ 1.34)		
ALCS of 20^th^ fraction during RT		0.086	3.01(0.86 ~ 10.54)		
ALCS of 1month after RT		0.045	0.46 (0.21 ~ 0.98)	0.041	0.44(0.20~ 0.97)
Decreased lymphocytes		0.038	0.45 (0.22 ~ 0.96)	0.041	0.46(0.22~ 0.97)
Elevated lymphocytes		0.006	0.34 (0.16 ~ 0.73)	0.005	0.33(0.15~ 0.72)

ECOG, The Eastern Cooperative Oncology Group Performance Status (ECOG) score; ALCS, absolute lymphocyte counts; RT, radiotherapy; OR, Odds Ratio; CI, confidence interval.

The optimal cutoff values for ALCs at 1 month after radiotherapy (1.015 × 10^9^/L), ALC decrease (0.71 × 10^9^/L), and ALC increase (0.305 × 10^9^/L) were determined using ROC curve analysis in SPSS (**Supplementary Figure 1**). Baseline characteristics of the two groups were compared to confirm comparability (**Supplementary Tables 2–4**). Patients with ALCs > 1.015 × 10^9^/L at 1 month after radiotherapy, ALC decrease > 0.71 × 10^9^/L, or ALC increase > 0.305 × 10^9^/L showed significantly better OS (p < 0.05) (**Figure 2**).

### Changes of lymphocyte subsets before and 1 month after radiotherapy

**Figure 3:**
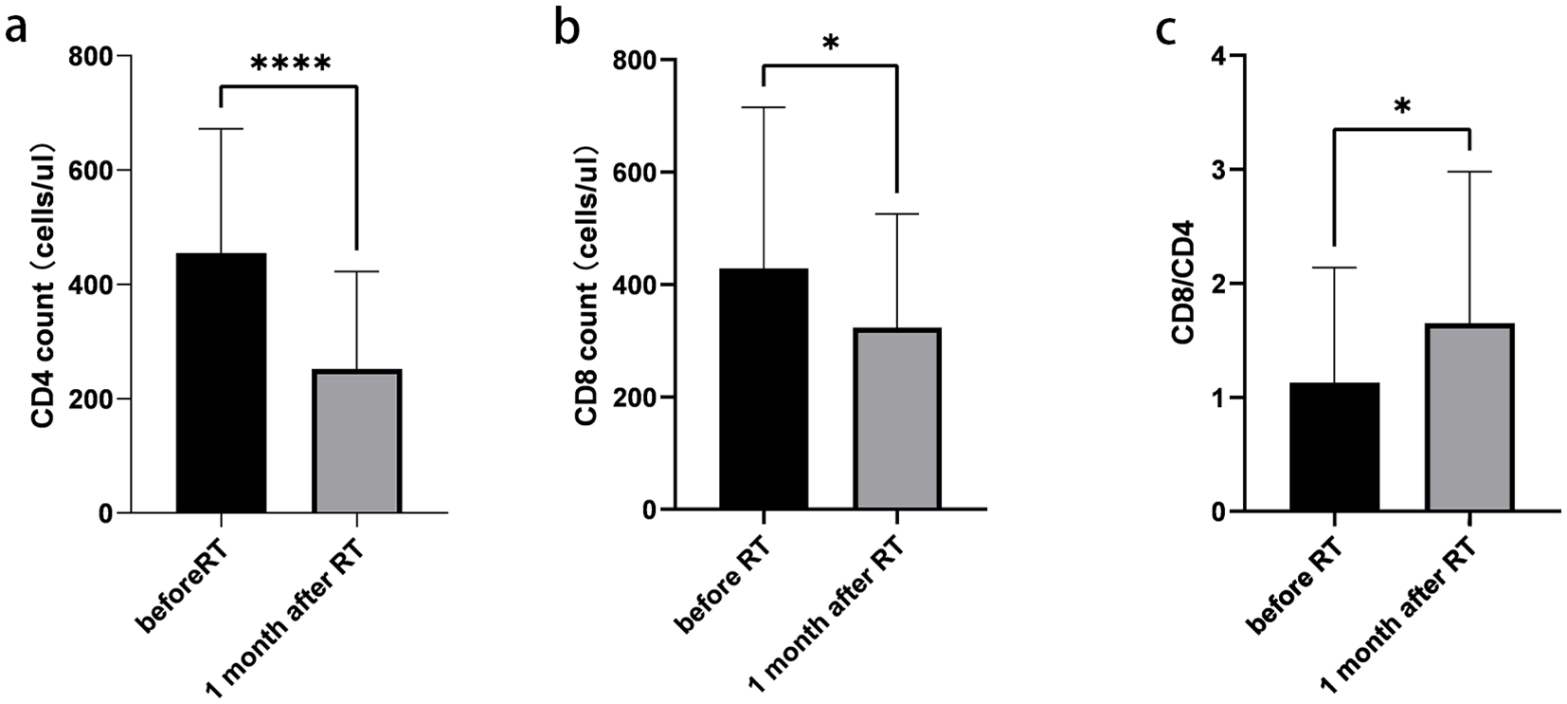
Changes of lymphocyte subsets before and 1 month after radiotherapy. **(a)** Absolute counts of CD4+ T cells. **(b)** Absolute counts of CD8+ T cells. **(c)** CD8+/CD4+ ratio. Data are presented as mean ± SD. *p < 0.05, ****p < 0.0001, paired t-test.

A total of 39 patients were assessed for lymphocyte subsets before and 1 month after radiotherapy. Detailed information on all lymphocyte subpopulations is provided in **Supplementary Table 5**. The absolute counts of CD4+ T cells, CD8+ T cells, and the CD8/CD4 ratio were measured before and 1 month after radiotherapy. Significant differences were observed in these parameters between the two time points (p < 0.05) (**Figure 3**).

### Relationship between changes in lymphocyte subsets and OS

ROC curve analysis was performed using the SPSS software to determine the optimal cutoff values. The cutoff values for CD4+, CD8+, and CD8/CD4 were 605/µL, 538/µL, and 0.79 before radiotherapy, and 140.5/µL, 230/µL, and 1.168 1 month after radiotherapy, respectively (**Supplementary Figure 2**).

**Figure 4:**
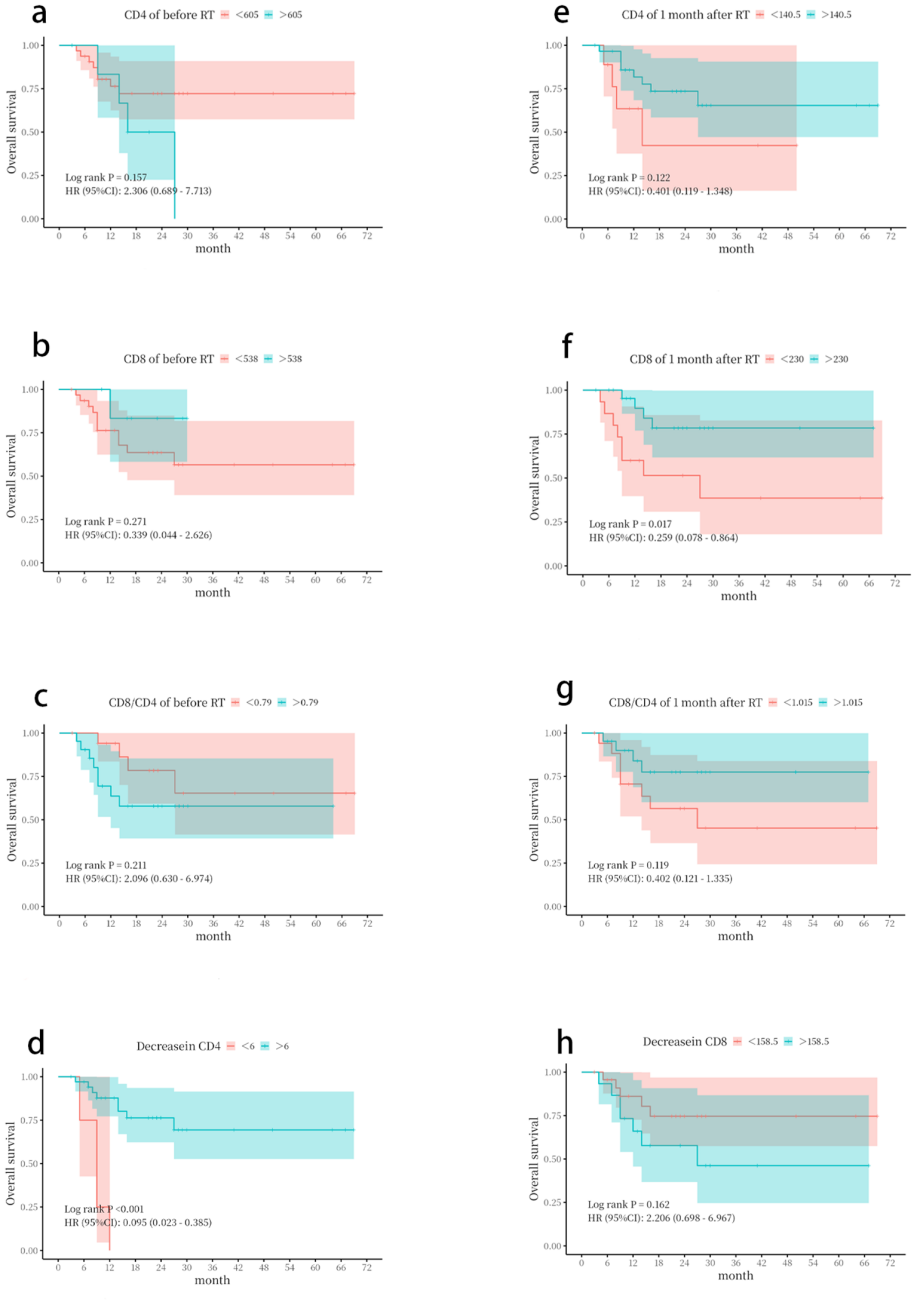
Kaplan–Meier survival curves based on lymphocyte subset parameters. Overall survival (OS) was stratified by **(a)** CD4+ T-cell count before radiotherapy. **(b)** CD8+ T-cell count before radiotherapy. **(c)** CD8+/CD4+ ratio before radiotherapy. **(d)** CD4+ T-cell count decrease. **(e)** CD4+ T-cell count at 1 month following radiotherapy. **(f)** CD8+ T-cell count at 1 month following radiotherapy. **(g)** CD8+/CD4+ ratio at 1 month following radiotherapy. **(h)** CD8+ T-cell count decrease. The optimal cut-off values for stratification were determined by receiver operating characteristic (ROC) curve analysis. p-Values were calculated using the log-rank test.

The Kaplan–Meier analysis showed that patients with an absolute CD8+ T-cell count > 230/µL or a CD4+ T-cell count decrease of <6/µL at 1 month after radiotherapy had significantly better OS (**Figure 4**).

## Discussion

The emergence of ICIs in recent years has significantly changed the treatment paradigm for NSCLC patients worldwide (20). However, responses to anti-PD-1 inhibitor therapy have been inconsistent among NSCLC patients (21). Consequently, identifying which patients are most likely to respond favorably to anti-PD-1 inhibitors has become a major challenge. Yu Wang et al. reported that, in real-world studies (RWSs), the median interval between the completion of concurrent chemoradiotherapy and the initiation of durvalumab often exceeded 42 days, representing a notable deviation from the PACIFIC study protocol, which specified a maximum interval of 42 days (22). The main reason is the delay of consolidation immunotherapy due to adverse reactions. While durvalumab is standard per PACIFIC, this study used sintilimab, an anti-PD-1 inhibitor widely accessible in China post-national reimbursement druglist (NRDL) inclusion (2021). This reflects real-world practice during the study period (2018–2024), as supported by comparable efficacy observed in advanced NSCLC in the ORIENT-11 study (23). Furthermore, changes in the tumor immune microenvironment after chemoradiotherapy highlight the need for a simple and convenient predictor. Lymphocytes, which account for approximately 30% of total white blood cells in healthy individuals, are among the most radiation-sensitive cell subsets and play a crucial role as effector cells in anti-tumor immunity (24). As a reflection of the host’s immune status, lymphocyte levels are closely associated with responses to immune checkpoint inhibitors and OS in melanoma patients (25). Studies have indicated that ALCs are prognostic in various malignancies, including NSCLC. Low ALCs are associated with poor prognosis in lung, breast, and colorectal cancers (26). In NSCLC patients treated with nivolumab, ALCs can predict OS. Moreover, baseline ALCs, ALCs at 3 months, and changes in ALCs serve as dynamic biomarkers for predicting the efficacy of ICI treatment (27).

Adaptive radiotherapy (ART) is an image-guided technique for tumor treatment. Patients with NSCLC who have large tumor lesions and high radiosensitivity are particularly well-suited for ART. The radiotherapy dose range of 30–50 Gy delivered in 15–25 fractions may represent the optimal timing for implementing ART (28). Our previous research indicated that GTVs decreased by a median of 38.2% after receiving 42–44 Gy of radiotherapy in 20 fractions. By accounting for tumor and anatomical shifts, dosimetric parameters for organs at risk were notably reduced. Specifically, the average irradiation dose to the lungs decreased by 74.8 cGy, and the average dose to the esophagus was reduced by 183.1 cGy. Adaptive adjustments were also associated with a 3-percentage-point reduction in the probability of Grade 2 radiation pneumonitis and a 5-percentage-point reduction in the risk of Grade 2 radiation esophagitis (18). Additionally, the trajectory of ALCs during radiotherapy showed a general downward trend, followed by gradual recovery after radiotherapy. In our study, after 20 fractions of ART, ALC levels were 0.61 ± 0.80 × 10^9^/L, representing a decrease of 0.78 ± 0.94 × 10^9^/L from baseline. One month after radiotherapy, ALCs increased to 1.10 ± 0.52 × 10^9^/L, with an increase of 0.49 ± 0.92 × 10^9^/L. In comparison, van Rossum P.S.N. et al. reported that at the fourth week, ALCs decreased to 0.61 ± 0.80 × 10^9^/L, a reduction of 0.78 ± 0.94 × 10^9^/L from baseline (29). This slightly smaller reduction in our study may be attributed to the reduced irradiation volume achieved by adaptive radiotherapy, which could mitigate the extent of lymphocyte depletion. This observation warrants further investigation.

In our study, univariate and multivariate analyses showed that ALCs at 1 month after radiotherapy, and decreases and increases in ALCs were independent prognostic factors associated with significantly improved OS. Patients with ALCs > 1.015 × 10^9^/L at 1 month after radiotherapy, but not at baseline, demonstrated better OS (p < 0.05). The central question is how these peripheral blood measurements reflect the efficacy of consolidation immunotherapy acting within the tumor microenvironment (TME). The answer lies in the dynamic interplay between systemic and local anti-tumor immunity. Lymphocytes continuously recirculate between the blood, lymphoid organs, and peripheral tissues (30). Critically, studies have demonstrated a significant clonal overlap between T cells in peripheral blood and TILs, and the expansion of specific T-cell clones in the periphery can predict their subsequent infiltration into tumors (31, 32). Therefore, the early recovery of lymphocytes, particularly CD8+ T cells, in the blood post-CRT likely signifies a robust systemic immune reconstitution. This reconstituted peripheral immune reservoir is essential for supplying functional effector cells to the TME, thereby creating a favorable context for subsequent anti-PD-1 therapy to unleash a potent and durable anti-tumor response (33). The more pronounced association of lymphocyte parameters showed a stronger association with OS than with PFS. We posit that this dissociation reflects a fundamental distinction between the mechanisms of action of underlying immunotherapy and those of cytotoxic therapy. Specifically, immune reconstitution establishes long-term immunologic memory, thereby providing continuous surveillance against tumor recurrence (34). This sustained biological activity, which can control disease over extended periods and even after initial progression, is inherently better captured by OS than by PFS, which primarily measures the time to first radiographic progression. Our results thereby align with a growing body of evidence suggesting that biomarkers of a competent immune microenvironment are more strongly linked to ultimate survival outcomes than to early radiographic response metrics (35). In this context, post-CRT lymphocyte recovery emerges not merely as a predictor of initial response but as an indicator of a patient’s ability to establish durable disease control. This suggests that RT has a potential effect on the proliferation and activation of lymphocytes (36). Although radiotherapy can have an immunostimulatory effect by inducing neoantigens and danger signals that activate the immune system (37), it also exerts immunosuppressive effects, such as lymphopenia. The extent of these effects depends on the timing of blood sampling and lymphodepletion. Some studies have shown that these changes can recover within 2, 3, or 6 months, and up to 1 year post-treatment (37). Eckert et al. observed that during radiotherapy (RT), all immune cell subgroups, except regulatory T cells (Tregs), exhibited elevated proliferation rates, which normalized approximately 3 months after treatment (38). However, there are no studies demonstrating the extent to which lymphocytes can recover sufficiently to continue consolidation immunotherapy. Radiotherapy and immunotherapy also exhibit synergistic effects. Radiotherapy can enhance the efficacy of immunotherapy against tumors by promoting the release of damage-associated molecular patterns (DAMPs) and tumor-associated antigens (TAAs), optimizing the tumor immune microenvironment, and creating hypoxic conditions in tumor cells. ICIs can help overcome radiation resistance induced by radiotherapy. Conversely, ICIs activate T cells against tumor cells (39–41), normalize tumor vasculature, regulate the microenvironment, and increase radiosensitivity. The mechanisms underlying this synergy require further investigation (42).

In our study, patients with stage III non-small cell lung cancer receiving chemoradiotherapy combined with immunotherapy exhibited a decrease in ALCs during radiotherapy, which partially recovered 1 month after treatment. Significant decreases in absolute counts of CD4+ and CD8+ T cells, as well as in the CD8+/CD4+ ratio, were observed at 1 month post-radiotherapy compared to baseline, with CD4+ T cells showing a more pronounced decline. Patients with absolute CD8+ T-cell counts > 230/µL and CD4+ T cell decreases < 6/µL at 1 month after radiotherapy demonstrated better OS. In one study, the levels of CD3+, CD4+, CD4+/CD8+, and CD19+ cells in the peripheral blood of lung cancer patients before radiotherapy were significantly lower than those in healthy controls (p < 0.05) (43), which is consistent with our findings. The effect of radiotherapy on the immune microenvironment appears to be a double-edged sword: it can activate anti-tumor immunity, but it also induces lymphocyte depletion and immunosuppression. However, radiotherapy may preferentially eliminate tumor-resistant immune cells and help restore the immune microenvironment. The faster recovery of CD8+ T cells compared to CD4+ T cells results in an increased CD8/CD4 ratio, which may enhance the efficacy of immunotherapy. These results warrant further validation in prospective clinical studies.

The primary limitation of this study is its single-center retrospective design and relatively small sample size, which may introduce potential information bias. Therefore, to further validate our findings, a prospective, multicenter study with a larger cohort is warranted. Additionally, future research should include a more comprehensive analysis of lymphocyte subtypes, extending beyond CD8+ and CD4+ cells. Another potential focus is whether targeted modulation of lymphocyte levels could further enhance the survival benefits of chemoradiotherapy followed by immunotherapy.

## Conclusion

This study demonstrated that ALCs or CD8+ T-cell counts measured 1 month after chemoradiotherapy can serve as effective prognostic predictors in NSCLC patients receiving chemoradiotherapy followed by consolidation immunotherapy.

## Data Availability

The raw data supporting the conclusions of this article will be made available by the authors, without undue reservation.

## Supplementary material

Supplementary Figure 1 Receiver operating characteristic (ROC) curves for absolute lymphocyte count parameters. ROC curves analyzing the predictive power of absolute lymphocyte counts for overall survival at different time points.

Supplementary Figure 2 Receiver operating characteristic (ROC) curves for lymphocyte subset parameters. ROC curves analyzing the predictive power of various lymphocyte subset parameters for overall survival: **(A)** CD4+ T cell count before radiotherapy. **(B)** CD8+ T cell count before radiotherapy. **(C)** CD8+/CD4+ ratio before radiotherapy. **(D)** CD4+ T cell count decrease. **(E)** CD4+ T cell count at one month following radiotherapy. **(F)** CD8+ T cell count at one month following radiotherapy. **(G)** CD8+/CD4+ ratio at one month following radiotherapy. **(H)** CD8+ T cell count decrease.
