# Supplementary figures and images for: Effectiveness and mechanisms of lymphocytes at different time points in predicting consolidation immunotherapy following adaptive chemoradiotherapy in locally advanced non-small cell lung cancer
Source: Front Oncol. 2026 Jan 9;15:1683430. doi: 10.3389/fonc.2025.1683430 (PMC12827176; doi:10.3389/fonc.2025.1683430)

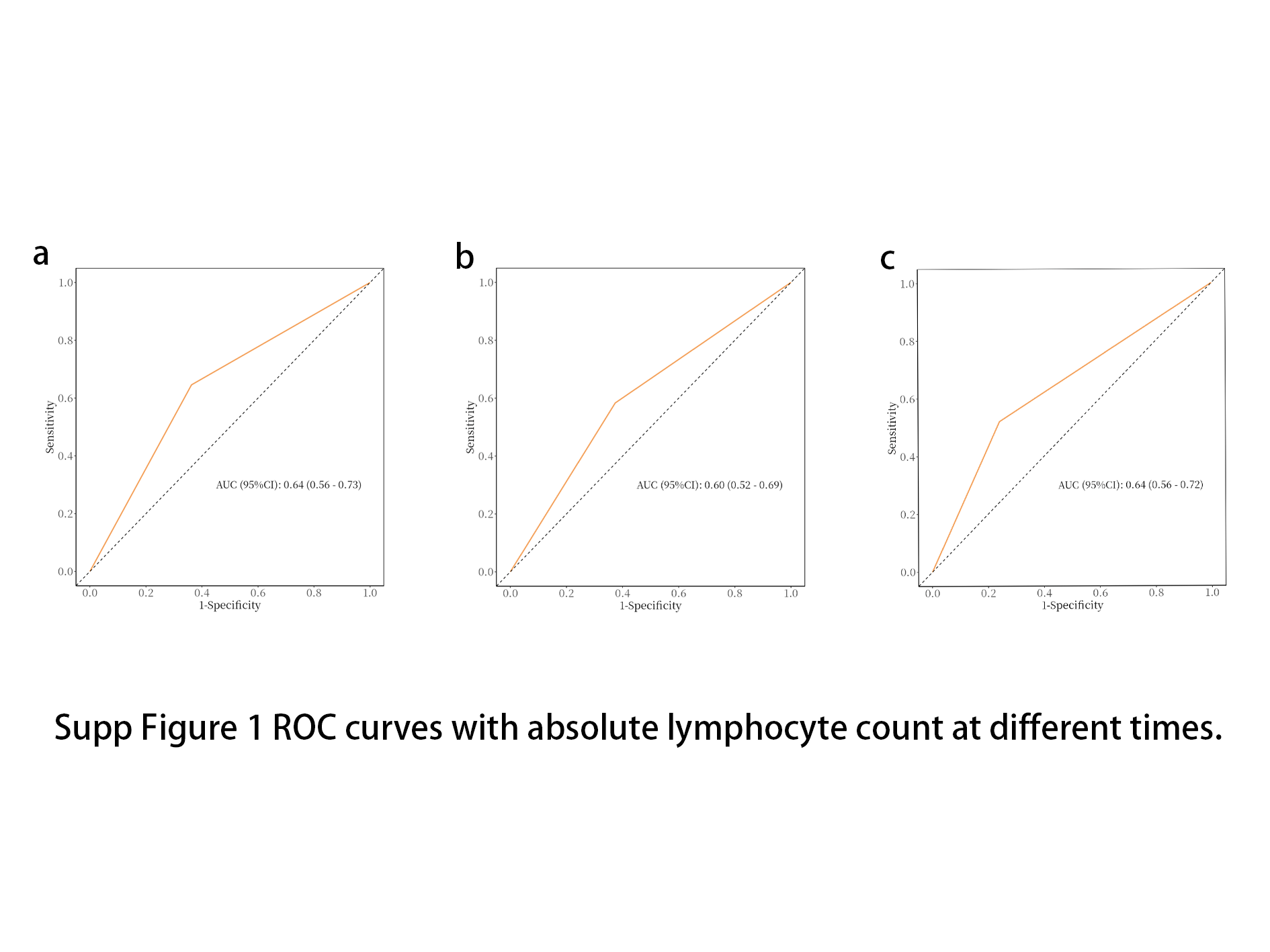

Supplement: Supplementary Figure 1 — Receiver operating characteristic (ROC) curves for absolute lymphocyte count parameters. ROC curves analyzing the predictive power of absolute lymphocyte counts for overall survival at different time points. [file Image1.tif]

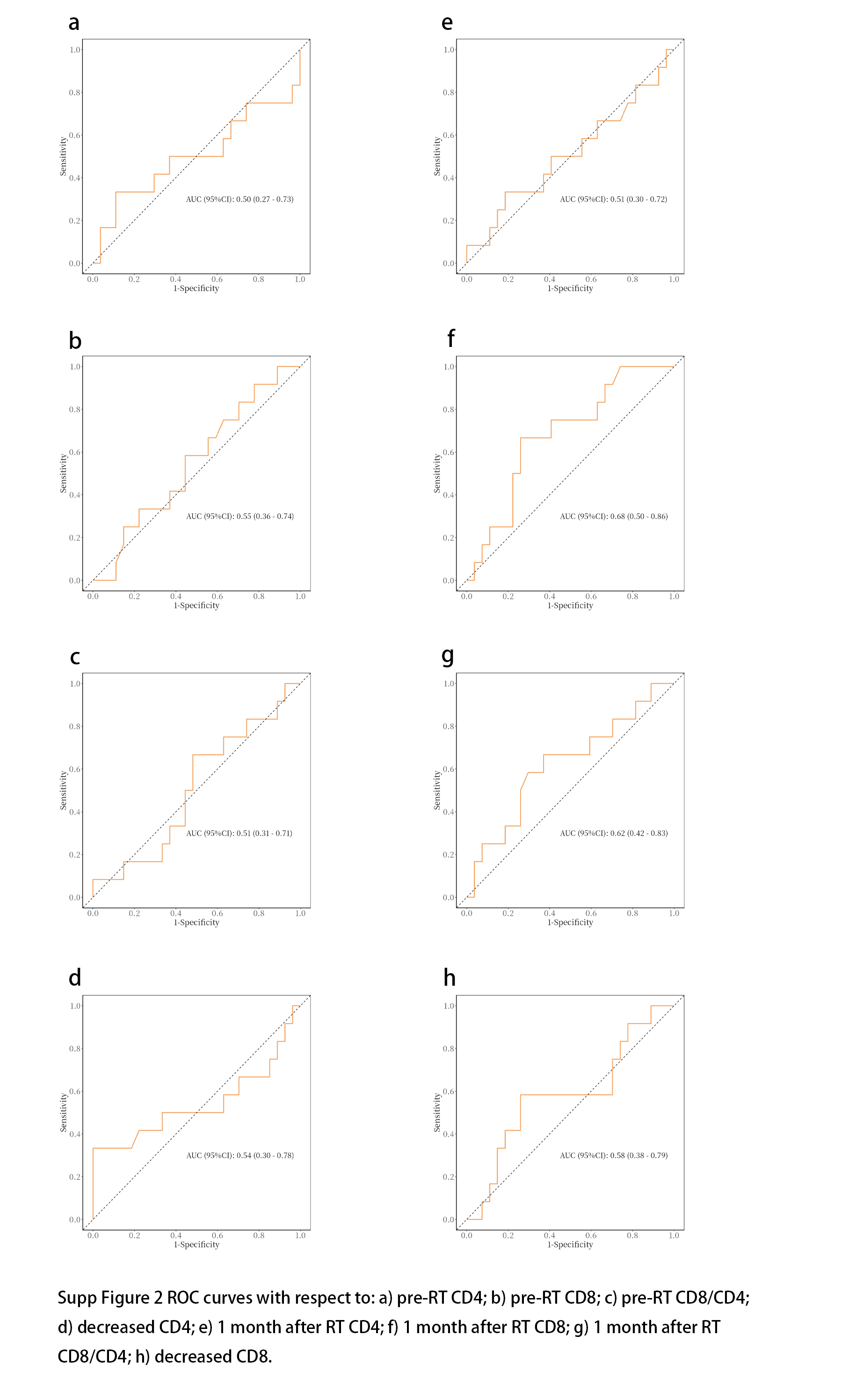

Supplement: Supplementary Figure 2 — Receiver operating characteristic (ROC) curves for lymphocyte subset parameters. ROC curves analyzing the predictive power of various lymphocyte subset parameters for overall survival: (A) CD4+ T cell count before radiotherapy. (B) CD8+ T cell count before radiotherapy. (C) CD8+/CD4+ ratio before radiotherapy. (D) CD4+ T cell count decrease. (E) CD4+ T cell count at one month following radiotherapy. (F) CD8+ T cell count at one month following radiotherapy. (G) CD8+/CD4+ ratio at one month following radiotherapy. (H) CD8+ T cell count decrease. [file Image2.tif]
