# Supplementary material for: Effectiveness and mechanisms of lymphocytes at different time points in predicting consolidation immunotherapy following adaptive chemoradiotherapy in locally advanced non-small cell lung cancer
Source: Front Oncol. 2026 Jan 9;15:1683430. doi: 10.3389/fonc.2025.1683430 (PMC12827176; doi:10.3389/fonc.2025.1683430)
Supplement: Supplementary file 3 [file Table1.docx]

| Suppl Table 1 Univariate cox analysis of progression free survival | | | |
| --- | --- | --- | --- |
| Variables | | OR (95%CI) | *P* |
| Age | | 0.98 (0.95 ~ 1.02) | 0.449 |
| Gender | Male | 1.00 (Reference) |  |
|  | Female | 0.27 (0.06 ~ 1.27) | 0.098 |
| ECOG | 0 | 1.00 (Reference) |  |
|  | 1 | 0.63 (0.28 ~ 1.42) | 0.265 |
| Smoking | NO | 1.00 (Reference) |  |
|  | YES | 1.09 (0.49 ~ 2.44) | 0.837 |
| T | 1 | 1.00 (Reference) |  |
|  | 2 | 0.95 (0.26 ~ 3.50) | 0.944 |
|  | 3 | 1.10 (0.28 ~ 4.37) | 0.888 |
|  | 4 | 1.36 (0.41 ~ 4.49) | 0.610 |
| N | 1 | 1.00 (Reference) |  |
|  | 2 | 0.67 (0.22 ~ 2.04) | 0.477 |
|  | 3 | 1.38 (0.38 ~ 4.91) | 0.624 |
| Stage | ⅢA | 1.00 (Reference) |  |
|  | ⅢB | 2.16 (0.95 ~ 4.89) | 0.065 |
|  | ⅢC | 1.60 (0.53 ~ 4.83) | 0.405 |
| Pathologic | Squamous | 1.00 (Reference) |  |
|  | Adenocarcinoma | 0.38 (0.06 ~ 2.37) | 0.297 |
|  | Other | 0.80 (0.21 ~ 3.08) | 0.742 |
| Therapy Method | Concurrent Chemoradiotherapy | 1.00 (Reference) |  |
|  | Sequential Chemoradiotherapy | 1.05 (0.50 ~ 2.20) | 0.902 |
| ALCS before RT （10^9^ /L） | |  |  |
| ALCS of 20^th^ Fraction during RT（10^9^ /L） | |  |  |
| ALCS of 1 month after RT（10^9^ /L） | | 1.54 (0.72 ~ 3.27) | 0.264 |
| Decreased lymphocytes（10^9^ /L） | | 1.65 (0.77 ~ 3.53) | 0.199 |
| Increased lymphocytes（10^9^ /L） | | 1.03 (0.47 ~ 2.26) | 0.940 |
| ECOG:The Eastern Cooperative Oncology Group Performance Status (ECOG) score. ALCS: absolute lymphocyte counts. RT: radiotherapy. OR: Odds Ratio. CI: confidence interval. | | | |

| Suppl Table 2 Baseline characteristics on account of patients ALCS 1 month after radiotherapy | | | | |
| --- | --- | --- | --- | --- |
| Variables | | ＞1.015 (n = 75) | ≤1.015 (n = 64) | *P* |
| Age (M) |  | 69.00 (58.50, 73.50) | 68.00 (57.00, 80.00) | 0.225 |
| Gender, n(%) | Female | 4 (5.33) | 3 (4.69) | 1.000 |
|  | Male | 71 (94.67) | 61 (95.31) |  |
| ECOG, n(%) | 0 | 53 (70.67) | 49 (76.56) | 0.433 |
|  | 1 | 22 (29.33) | 15 (23.44) |  |
| Smoking, n(%) | YES | 53 (70.67) | 45 (70.31) | 0.964 |
|  | NO | 22 (29.33) | 19 (29.69) |  |
| T, n(%) | 1 | 7 (9.33) | 9 (14.06) | 0.669 |
|  | 2 | 15 (20.00) | 16 (25.00) |  |
|  | 3 | 14 (18.67) | 10 (15.62) |  |
|  | 4 | 39 (52.00) | 29 (45.31) |  |
| N, n(%) | 1 | 12 (16.00) | 8 (12.50) | 0.301 |
|  | 2 | 45 (60.00) | 33 (51.56) |  |
|  | 3 | 18 (24.00) | 23 (35.94) |  |
| Stage, n(%) | ⅢA | 26 (34.67) | 22 (34.38) | 0.662 |
|  | ⅢB | 39 (52.00) | 30 (46.88) |  |
|  | ⅢC | 10 (13.33) | 12 (18.75) |  |
| Pathologic, n(%) | Squamous | 62 (82.67) | 55 (85.94) | 0.301 |
|  | Adenocarcinoma | 6 (8.00) | 7 (10.94) |  |
|  | Other | 7 (9.33) | 2 (3.12) |  |
| Therapy Method, n(%) | Concurrent Chemoradiotherapy | 33 (44.00) | 30 (46.88) | 0.734 |
|  | Sequential Chemoradiotherapy | 42 (56.00) | 34 (53.12) |  |
| ECOG:The Eastern Cooperative Oncology Group Performance Status (ECOG) score. ALCS: absolute lymphocyte counts. RT: radiotherapy. | | | | |

| Suppl Table 3 Baseline characteristics on account of patients decreased ALCS | | | | |
| --- | --- | --- | --- | --- |
| Variables | | ＞0.71 (n = 77) | ≤0.71 (n = 62) | P |
|  |  |  |  |  |
| Age (year) | | 69.00 (63.00, 80.00) | 67.00 (55.00, 71.00) | 0.036 |
| Gender, n(%) | Female | 3 (3.90) | 4 (6.45) | 0.768 |
|  | Male | 74 (96.10) | 58 (93.55) |  |
| ECOG, n(%) | 0 | 53 (68.83) | 49 (79.03) | 0.176 |
|  | 1 | 24 (31.17) | 13 (20.97) |  |
| Smoking, n(%) | YES | 53 (68.83) | 45 (72.58) | 0.630 |
|  | NO | 24 (31.17) | 17 (27.42) |  |
| T, n(%) | 1 | 7 (9.09) | 9 (14.52) | 0.647 |
|  | 2 | 16 (20.78) | 15 (24.19) |  |
|  | 3 | 15 (19.48) | 9 (14.52) |  |
|  | 4 | 39 (50.65) | 29 (46.77) |  |
| N, n(%) | 1 | 13 (16.88) | 7 (11.29) | 0.595 |
|  | 2 | 43 (55.84) | 35 (56.45) |  |
|  | 3 | 21 (27.27) | 20 (32.26) |  |
| Stage, n(%) | ⅢA | 28 (36.36) | 20 (32.26) | 0.749 |
|  | ⅢB | 36 (46.75) | 33 (53.23) |  |
|  | ⅢC | 13 (16.88) | 9 (14.52) |  |
| Pathologic, n(%) | Squamous | 66 (85.71) | 51 (82.26) | 0.393 |
|  | Adenocarcinoma | 5 (6.49) | 8 (12.90) |  |
|  | Other | 6 (7.79) | 3 (4.84) |  |
| Therapy Method, n(%) | Concurrent Chemoradiotherapy | 34 (44.16) | 29 (46.77) | 0.758 |
|  | Sequential Chemoradiotherapy | 43 (55.84) | 33 (53.23) |  |
| ECOG:The Eastern Cooperative Oncology Group Performance Status (ECOG) score. ALCS: absolute lymphocyte counts. RT: radiotherapy. | | | | |

| Suppl Table 4 Baseline characteristics on account of patients increased ALCS | | | | |
| --- | --- | --- | --- | --- |
| Variables | | ＞0.305 (n = 92) | ≤0.305 (n = 47) | *P* |
|  |  |  |  |  |
| Age, (M) | | 69.00 (58.75, 73.00) | 67.00 (55.50, 80.0) | 0.294 |
| Gender, n(%) | Female | 5 (5.43) | 2 (4.26) | 1.000 |
|  | Male | 87 (94.57) | 45 (95.74) |  |
| ECOG, n(%) | 0 | 63 (68.48) | 39 (82.98) | 0.067 |
|  | 1 | 29 (31.52) | 8 (17.02) |  |
| Smoking, n(%) | YES | 62 (67.39) | 36 (76.60) | 0.260 |
|  | NO | 30 (32.61) | 11 (23.40) |  |
| T, n(%) | 1 | 8 (8.70) | 8 (17.02) | 0.150 |
|  | 2 | 15 (16.30) | 16 (34.04) |  |
|  | 3 | 20 (21.74) | 4 (8.51) |  |
|  | 4 | 49 (53.26) | 19 (40.43) |  |
| N, n(%) | 1 | 15 (16.30) | 5 (10.64) | 0.549 |
|  | 2 | 52 (56.52) | 26 (55.32) |  |
|  | 3 | 25 (27.17) | 16 (34.04) |  |
| Stage, n(%) | ⅢA | 30 (32.61) | 18 (38.30) | 0.466 |
|  | ⅢB | 45 (48.91) | 24 (51.06) |  |
|  | ⅢC | 17 (18.48) | 5 (10.64) |  |
| Pathologic, n(%) | Squamous | 78 (84.78) | 39 (82.98) | 0.495 |
|  | Adenocarcinoma | 7 (7.61) | 6 (12.77) |  |
|  | Other | 7 (7.61) | 2 (4.26) |  |
| Therapy Method, n(%) | Concurrent Chemoradiotherapy | 37 (40.22) | 26 (55.32) | 0.091 |
|  | Sequential Chemoradiotherapy | 55 (59.78) | 21 (44.68) |  |
| ECOG:The Eastern Cooperative Oncology Group Performance Status (ECOG) score. ALCS: absolute lymphocyte counts. RT: radiotherapy. | | | | |
|  | | | | |

| Suppl Table 5 Baseline Characteristics of Patients by lymphocyte subsets (n=39) | | |
| --- | --- | --- |
| Variables | | Total (n = 39) |
| Age (year) | | 69.00 (48, 80) |
| Gender  n(%) | Female | 2 (5.13) |
|  | Male | 37 (94.87) |
| ECOG  n(%) | 0 | 28 (71.79) |
|  | 1 | 11 (28.21) |
| Smoking  n(%) | YES | 27 (69.23) |
|  | NO | 12 (30.77) |
| T  n(%) | 1 | 3 (7.69) |
|  | 2 | 11 (28.21) |
|  | 3 | 5 (12.82) |
|  | 4 | 20 (51.28) |
| N  n(%) | 1 | 5 (12.82) |
|  | 2 | 23 (58.97) |
|  | 3 | 11 (28.21) |
| Stage  n(%) | ⅢA | 15 (38.46) |
|  | ⅢB | 17 (43.59) |
|  | ⅢC | 7 (17.95) |
| Pathologic  n(%) | Squamous | 33 (84.62) |
|  | Adenocarcinoma | 3 (7.69) |
|  | Other | 3 (7.69) |
| Therapy Method  n(%) | Concurrent Chemoradiotherapy | 22 (56.41) |
|  | Sequential Chemoradiotherapy | 17 (43.59) |
| CD4 before RT (Mean ± SD)（**/**µL） | | 454.48 ± 217.83 |
| CD8 before RT (Mean ± SD)（**/**µL） | | 428.49 ± 286.56 |
| CD8/CD4 before RT(Mean ± SD)（**/**µL） | | 1.13 ± 1.00 |
| Decreased CD4 (Mean ± SD)（**/**µL） | | 202.49 ± 165.53 |
| CD4 of 1month after RT (Mean ± SD)（**/**µL） | | 251.99 ± 170.32 |
| CD8 of 1month after RT (Mean ± SD)（**/**µL） | | 323.62 ± 201.81 |
| CD8/CD4 of 1month after RT (Mean ± SD)（**/**µL） | | 1.65 ± 1.33 |
| Decreased CD8 (Mean ± SD)（**/**µL） | | 104.87 ± 263.91 |
| ECOG:The Eastern Cooperative Oncology Group Performance Status (ECOG) score. ALCS: absolute lymphocyte counts.RT: radiotherapy. SD: standard deviation | | |
